# Late-onset superior mesenteric artery syndrome four years following scoliosis surgery – a case report

**DOI:** 10.1051/sicotj/2015010

**Published:** 2015-06-15

**Authors:** Nariman Abol Oyoun, Muayad Kadhim, John P. Dormans

**Affiliations:** 1 Department of Orthopaedic Surgery, Assiut University Hospital Assiut 71526 Egypt; 2 Division of Orthopaedic Surgery, The Children’s Hospital of Philadelphia 3401 Civic Center Boulevard Philadelphia PA 19104 USA

**Keywords:** Superior Mesenteric Artery, SMA Syndrome, Scoliosis, Spinal fusion

## Abstract

*Background*: Superior mesenteric artery (SMA) syndrome has been reported as an uncommon condition of external vascular compression of the SMA particularly after rapid weight loss, body casts, or after corrective surgery for spinal deformities, usually within the first few weeks after surgery.

*Methods*: This is a retrospective report of a case of a non-verbal autistic female patient who started to develop SMA syndrome at the age of 16, 4 years after posterior spinal fusion surgery for scoliosis. She was treated conservatively by increasing oral caloric intake, which resulted in increased body weight and relief of symptoms.

*Results*: Seen at 10 years’ follow up, the patient is doing well, and is functional within the limits of her suboptimal cognitive and verbal conditions. She maintains good trunk balance with solid spinal fusion and intact instrumentation at latest follow up.

*Conclusion*: Spinal surgeons should maintain a high index of suspicion for diagnosis of SMA syndrome even years after scoliosis surgery, especially for patients with communication problems, like the case we present here. Appropriate conservative measures can succeed in relieving the symptoms, increasing body weight, and preventing complications including the risk of death.

## Introduction

Superior mesenteric artery syndrome (SMAS) is an uncommon condition of the gastrointestinal (GI) tract with external vascular compression of the third part of the duodenum causing partial or complete obstruction secondary to decreased aortomesenteric angle and distance to 6–16° and 2–8 mm, respectively [1–3]. It has been described in patients after rapid weight loss and after corrective surgery of spinal deformities [4], either with instrumentation or with application of a body cast [2, 5, 6], usually during the first postoperative week [7–10]. It has also been reported following a spica cast [11].

We present here a case of SMAS, where the symptoms ensued 4 years after surgery.

## Case report

**Figure 1.**
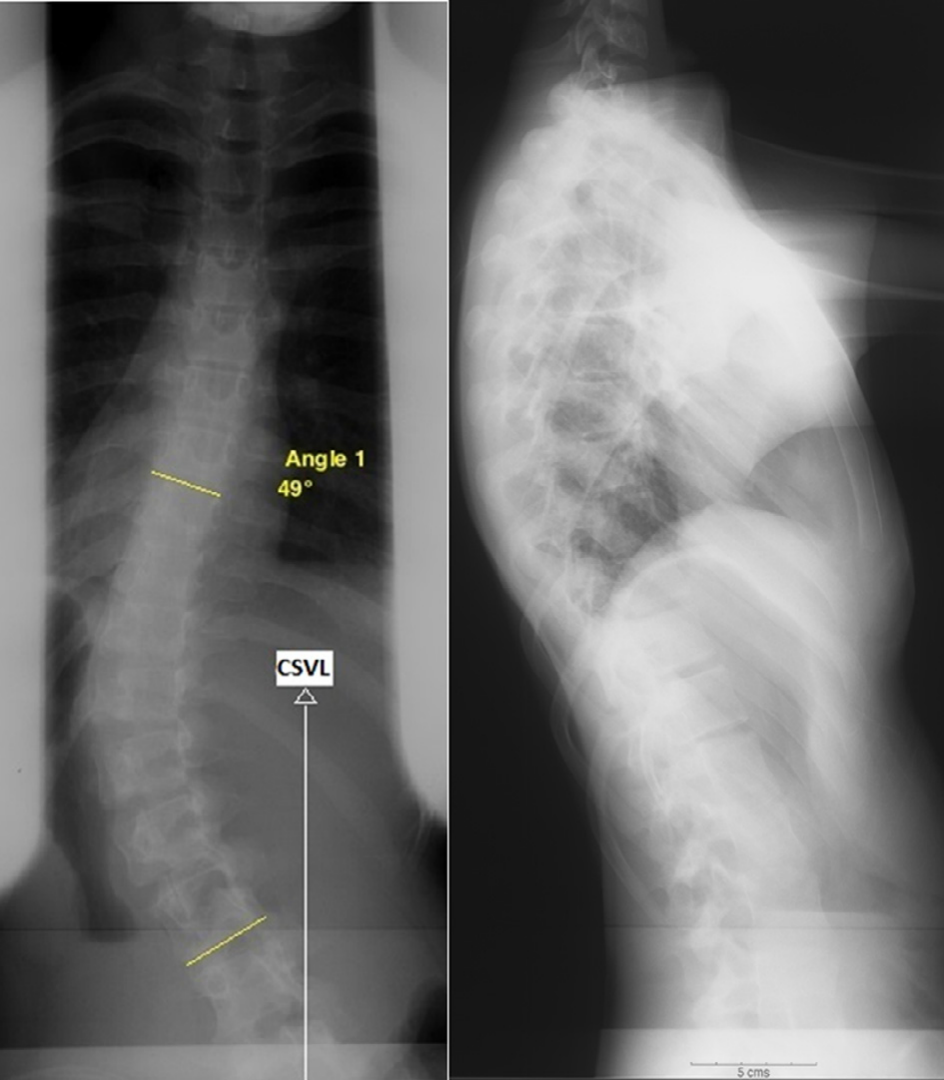
Preoperative spine radiographs showing scoliosis of 49° and the center sacral vertical line (CSVL) falling medial to the apical lumbar vertebra, a C lumbar modifier [34].

**Figure 2.**
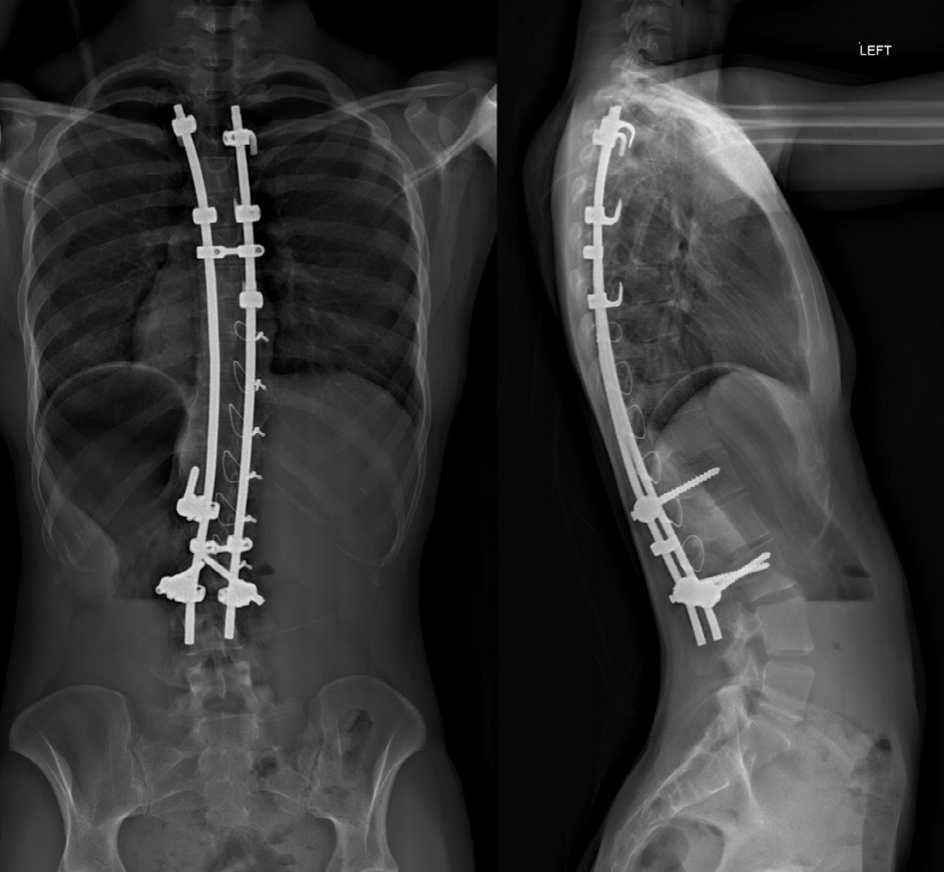
Latest follow-up radiographs 10 years after surgery.

A 12-year-old autistic, nonverbal, and developmentally delayed female, 2 years post-menarchal and underweight (height 155 cm, weight 36.2 kg, and BMI 14.97 kg/m^2^), underwent instrumented posterior spinal fusion for progressive scoliosis (Figures 1 and 2).

**Figure 3.**
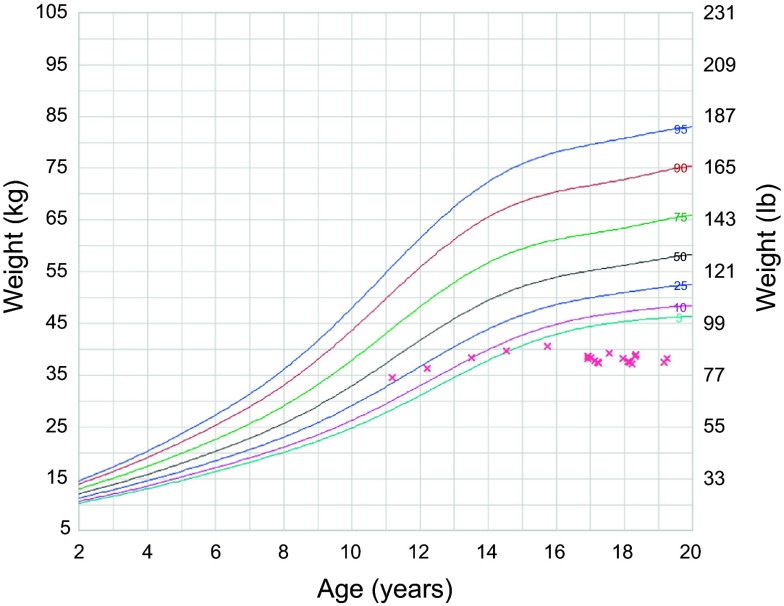
Weight for age chart of the patient showing consistent loss of weight captured around the age of 17, 5 years after surgery.

**Figure 4.**
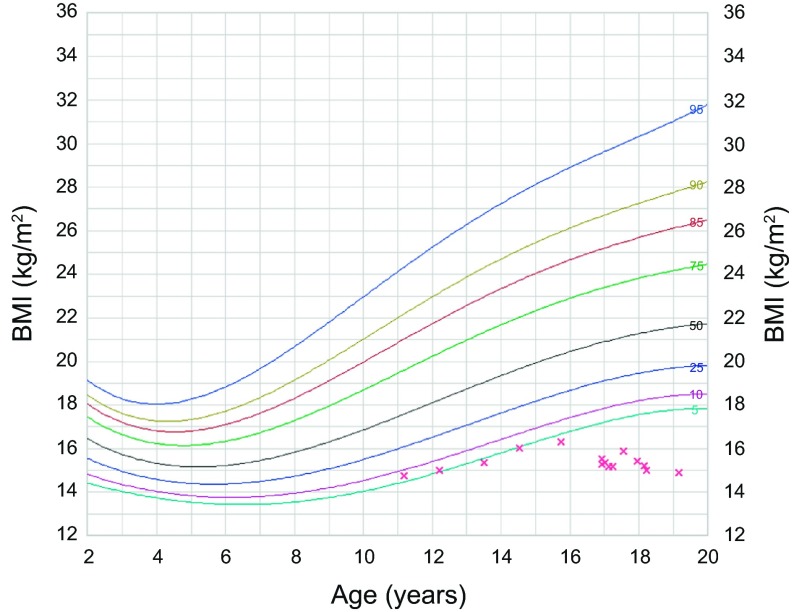
BMI for age chart of the patient showing generally low values with a drop captured around the age of 17, 5 years after surgery.

**Figure 5.**
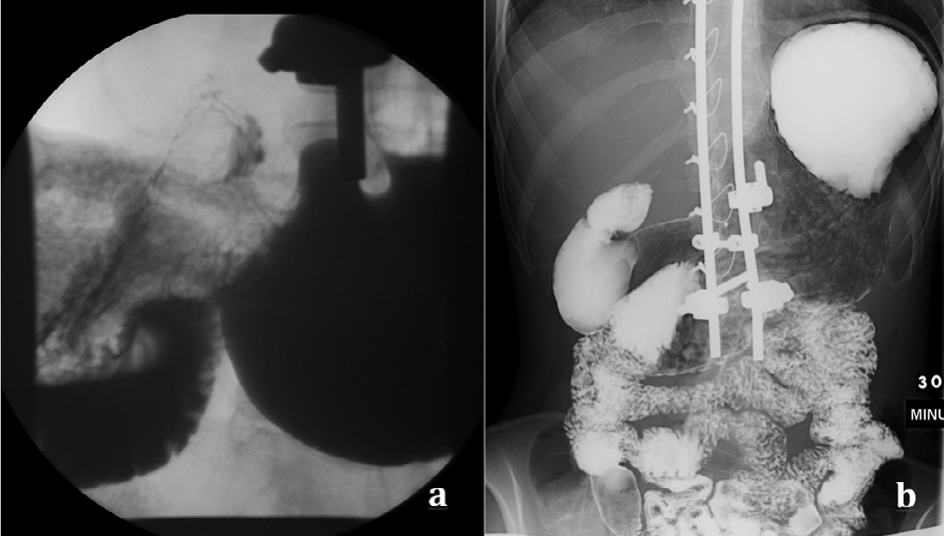
Barium Upper GI Study showing dilation of the stomach, 1st and 2nd parts of the duodenum. (a) Contrast not crossing to the left of the midline immediately after ingestion, (b) partial obstruction allowing some contrast into the jejunum later on.

Two years after the surgery, the patient showed increased agitation, anxiety, and autistic behavior. Whether these symptoms heralded or were possibly part of the clinical presentation of her later diagnosed SMAS cannot be confirmed. There was no vomiting or blood in stools, but it was difficult to determine whether she had abdominal pain. She had normal bowel movements and was “potty-trained”. Five years after surgery she was evaluated by a GI specialist for consistent weight loss over a year (Figures 3 and 4). Workup ruled out metabolic disorders, cystic fibrosis, and poor caloric intake with sweat test, serum amino acids, and urine organic acids within normal. Upper GI barium study showed immediate failure of the contrast to cross to the left side of the spine, but crossing minutes later with the incomplete nature of the obstruction (Figure 5), and to-and-fro motility between the third and second portions of the duodenum, consistent with SMAS. The diagnosis being confirmed, the gastroenterologist started the patient on a nutrition diet, increasing caloric intake to 2000–3000 Cal/day. Her symptoms improved and she gained some weight, but has always maintained a BMI below 16 kg/m^2^.

Ten years after surgery, with a small appetite, the patient seems to have enough energy and menstruates regularly. She works Fridays and goes outdoors with an aide. Her latest radiographs (Figure 2) show good trunk balance and solid spinal fusion with intact instrumentation.

## Discussion

SMAS has been reported after scoliosis surgery with a generally low incidence (1–4.7%) [2, 8, 10, 12], possibly due to relative lengthening of the spine increasing tension and narrowing the aortomesenteric angle [4, 13]. Also known as Cast Syndrome, SMAS is an uncommon, but a known, complication after the application of body casts [2, 5, 6, 14]. SMAS has also been reported to happen unrelated to spine surgery or casts, secondary to weight loss due to TB Cachexia [15], anorexia nervosa [16, 17], and other morbidities [18–20]. SMAS becomes self-perpetuating with a cycle of vomiting leading to further weight loss [4], and may co-occur with anterior nutcracker syndrome, with compression of the left renal vein [3, 21].

Symptoms of SMAS typically develop within a few days following scoliosis surgery [7, 9, 10, 22, 23]. Two cases were reported with somewhat delayed onset; a 14-year-old with SMAS progressed rapidly to death 40 days after scoliosis correction with Harrington instrumentation and application of a body cast [24]. Another presented 45 days after anterior spinal arthrodesis with postoperative bracing, but was treated conservatively with a favorable outcome [9]. In this case report, our patient presented as late as 5 years after her scoliosis surgery, which, to our knowledge, has not been previously reported.

In this report, we present a case of SMA syndrome with a subtle and unclear clinical presentation. Departing from the typical symptoms of SMA syndrome, known to be vomiting (92.9%), abdominal pain (57.1%), distension (42.9%), bilious vomiting (35.7%) [25], and hypoactive bowel sounds (28.6%) [9, 25], our patient collectively suffered agitation, anxiety, increased autistic behavior, possible abdominal pain, and weight loss.

A staged procedure, the lumbar modifier of B or C as opposed to A, body mass index (BMI) < 25th percentile, and increased stiffness of the thoracic curve are the most predictive of the development of SMAS after spinal deformity correction [2].

Barium Upper Gastrointestinal (GI) series was the key to diagnosis in our patient after exclusion of other differential diagnoses. The traditional diagnostic method for SMAS is the barium upper GI series, with four diagnostic criteria [26–28]. Magnetic resonance angiography and CT are used to evaluate the aortomesenteric angle [29]. Endoscopic ultrasound could determine both the pulsatile character of the duodenal compression and the reduced aortomesenteric distance at the site of stenosis [29].

The lack of high indices of clinical suspicion can cause diagnostic delays [4, 25, 30] and complications e.g. esophageal stricture [31]. The task is more demanding in the setting of a nonverbal patient with autism and mental retardation as in the patient we report here, especially in the absence of a known triggering factor. Our patient had higher odds of developing SMAS with a preoperative BMI < 25th percentile and a C lumbar modifier of her curve (Figure 1). The diagnosis of SMAS was suspected by her gastroenterologist based on a high index of suspicion, given her preoperative low BMI, her curve characteristics, and her presentation with consistent weight loss, and was confirmed by the Upper GI barium study. It is not clear in this patient, whether SMAS was triggered by an initial loss of weight, or has, on the other hand, been multifactorial, facilitated by her earlier scoliosis surgery. The initial treatment for SMAS should aim at correction of electrolyte imbalances, stomach decompression, and nutritional support (nasojejunal feeds or total parenteral nutrition) [32]. Medical treatment is attempted for at least 6 weeks before surgery is considered [28]. Patients with chronic disease and malnutrition despite conservative treatment may require surgical intervention [3, 33]. In our patient, conservative treatment alone was successful in increasing body weight, and improving her symptoms.

## Conclusion

The importance of maintaining a high index of clinical suspicion for SMAS cannot be overemphasized, specifically in patients who are at higher risk and who are unable to communicate. Although the onset of SMAS reported after scoliosis surgery did not exceed a few weeks, our patient had onset of GI-related symptoms, in the form of weight loss, and possible abdominal pain as late as 4 years after surgery. SMAS should take its place on the list of differential diagnosis even in the presence of non-GI-related symptoms, especially in nonverbal patients, to avoid diagnostic delay and prevent complications.

## Conflict of interest

NA, MK and JPD declare no conflict of interest in relation with this paper.
